# Vertex collocation profiles: theory, computation, and results

**DOI:** 10.1186/2193-1801-3-116

**Published:** 2014-02-28

**Authors:** Ryan N Lichtenwalter, Nitesh V Chawla

**Affiliations:** Interdisciplinary Center for Network Science and Applications (iCeNSA), The University of Notre Dame, 384 Nieuwland Hall, 46556 Notre Dame, USA; Department of Computer Science, The University of Notre Dame, 384 Fitzpatrick Hall, 46556 Notre Dame, USA

**Keywords:** Link prediction, Network analysis, Graph theory, Isomorphism

## Abstract

We describe the vertex collocation profile (VCP) concept. VCPs provide rich information about the surrounding local structure of embedded vertex pairs. VCP analysis offers a new tool for researchers and domain experts to understand the underlying growth mechanisms in their networks and to analyze link formation mechanisms in the appropriate sociological, biological, physical, or other context. The same resolution that gives the VCP method its analytical power also enables it to perform well when used to accomplish link prediction. We first develop the theory, mathematics, and algorithms underlying VCPs. We provide timing results to demonstrate that the algorithms scale well even for large networks. Then we demonstrate VCP methods performing link prediction competitively with unsupervised and supervised methods across different network families. Unlike many analytical tools, VCPs inherently generalize to multirelational data, which provides them with unique power in complex modeling tasks. To demonstrate this, we apply the VCP method to longitudinal networks by encoding temporally resolved information into different relations. In this way, the transitions between VCP elements represent temporal evolutionary patterns in the longitudinal network data. Results show that VCPs can use this additional data, typically challenging to employ, to improve predictive model accuracies. We conclude with our perspectives on the VCP method and its future in network science, particularly link prediction.

## Introduction

A vertex collocation profile (VCP) is a vector describing all isomorphically distinct collocations of two vertices within all possible isomorphism classes of three or more vertices. Just like ordinary isomorphism concepts, VCPs generalize naturally to directed, multirelational structures. VCPs are superficially similar to both local triangle counting (Becchetti et al. 2010; Davis et al. 2011) and motif analysis (Milo et al. 2002), but the theory and methods presented here are distinguished even at a superficial level by non-trivial generalization to structures encompassing direction, multiple relations, and any number of vertices. In detailed study, VCPs are strikingly differentiated by their incorporation of isomorphic equivalence and vertex pair collocation.

Among other tasks, VCPs are particularly suited to link prediction, since links are described by two vertices of interest. Link prediction is the task of inferring links in a graph *G*_*t*+1_ based on the observation of a graph *G*_*t*_. It may be that *t*+1 follows *t* in time, or it may be that *t*+1 represents some other modification of the graph such as including additional links from expensive experiments (Getoor and Diehl 2005). Link prediction stated in this manner is a binary classification task in which the positive class comprises links that form and the negative class comprises links that do not form. Many existing link prediction models compress a selection of basic information in theoretically or empirically guided ways. By contrast VCPs restrictively represent the local topological information describing the embedding of the source and target vertices. VCPs also apply naturally to multirelational networks and can thereby encode a variety of additional information. This includes even continuous quantities, such as edge weights, via quantization. We demonstrate the effectiveness of VCPs in link prediction with purely structural information. Then we show how VCPs can incorporate temporal information, notoriously difficult to model effectively, to achieve further remarkable increases in performance.

### Related work

The fundamental idea of counting structural forms in a network to better understand its properties is not new. Motif analysis (Milo et al. 2002) analyzes the prevalence of structural forms in different types of networks to look for statistical signatures that provide information, such as taxonomic designations, about the network. Counting graphlets, connected induced subgraphs, to which a node is incident (Pržulj 2007) has already been used for a variety of descriptive and analytical purposes in networks. The method described in (Davis et al. 2011) counts partially closed multirelational triads anywhere in a graph and then computes the conditional transition probability of the triad closing with the link type of interest. Unpublished at the time of the original exposition of VCPs, the triad transition matrix (TTM) approach Juszczyszyn et al. (2011a, 2011b) is similar to (Davis et al. 2011) in that triadic transition probabilities are used to construct a score. VCPs are subtly but critically distinguished from (Davis et al. 2011) and TTM in the same manner: they do not employ transition probabilities either directly or indirectly but rather describe the collocation of vertex pairs within subgraph isomorphism classes embedded within the network topology.

The vanilla task of link prediction as we have defined it has a wealth of supporting literature, and it would be impossible to cover it all here. Liben-Nowell and Kleinberg offered a seminal guide to the topic in (Liben-Nowell and Kleinberg 2007). The work most directly related uses structural forms to inform transition probabilities Juszczyszyn et al. (2011a, 2011b). VCPs take advantage of the supervised classification framework in (Lichtenwalter et al. 2010), which involves undersampling, bootstrap aggregation, and random forest or random subspace classification algorithms by substituting the simple feature vector derived from topological analysis with the VCP. There are several other supervised classification frameworks (Al Hasan et al. 2006; Wang et al. 2007) for link prediction that use basic topological characteristics, unsupervised link predictors, node attributes, and other information to construct their feature vectors.

Since we venture to incorporate temporal information into our models, this research niche in link prediction also merits some discussion. In several studies of link prediction, authors have used longitudinal data, a series of events with timestamps of varying resolution that describes a network evolving through time (Liben-Nowell and Kleinberg 2007; Lichtenwalter et al. 2010; Murata and Moriyasu 2007; Scellato et al. 2011; Sharan and Neville 2008), to perform link prediction without actually using the temporal component in their models. One recent work attempts to answer the question of *when* rather than *if* a link will form in the future (Sun et al. 2012). Also distinct are works that perform general modeling based on temporal link analysis, which has a broader supporting literature (Amitay et al. 2004; Hill et al. 2006). It is much less common to actually consider the temporal dimension as a factor in constructing link prediction models due to the difficulty of creating representations and models that effectively incorporate time (Hill et al. 2006). (Liben-Nowell and Kleinberg 2007) briefly considers methods of treating temporally delineated periods. The only publications of which we are aware that directly address the problem of predicting links using temporal information are (Acar et al. 2009) and (Qiu et al. 2011). The authors of (Acar et al. 2009) achieve predictions by applying factorizations to third-order tensors, in which the complete link structure is contained in two of the dimensions as it exists at discrete temporal intervals indicated by the third dimension. Alternatively, they describe a method of collapsing the temporal information into a weighted two-dimensional link representation on which they subsequently perform matrix factorizations to achieve predictions. The authors of (Qiu et al. 2011) extend the framework proposed in (Lichtenwalter et al. 2010) to incorporate two compressed features extracted from the longitudinal data, which they term *activeness* and *recency*. In (Juszczyszyn et al. 2011a) and (Juszczyszyn et al. 2011b), the authors use the mean value of triad transition matrices from multiple time windows, and thus they do not actually benefit from temporal changes in network dynamics evident in the underlying longitudinal information. To our knowledge, ours is the first work to use multirelational models to encode temporal information for the purpose of performing data-driven predictions with machine learning algorithms. We employ VCPs to accomplish this. By encoding temporal information as multiple edge types in a multirelational graph representation, we can take advantage of the power of VCPs as a sophisticated multirelational modeling technique to incorporate trends in network evolution directly into our models.

### Contributions

This work offers several improved and expanded treatments of vertex collocation profiles compared to our work in (Lichtenwalter and Chawla 2012). In addition to a significantly generalized and more formal theoretical treatment, this work provides the following specific areas of new coverage. It generalizes the formulation of vertex collocation profiles to unify handling of undirected multirelational and directed multirelational networks. Simultaneously, it expands upon the original limited treatment of directionality so that the theoretical coverage of directed structures is as complete and rigorous as for undirected structures. We offer a formal definition of graph isomorphism and use it to provide and elucidate an equally formal definition of vertex collocation profiles. Accompanying this theoretical generalization are new source code offerings that allow users almost unrestricted freedom in scaling their computations to large, highly multirelational networks. We overcame some of the complexities of combinatoric explosion faced in highly multirelational networks by opting for memoized dynamic computation of isomorphisms. This has allowed us to apply vertex collocation profiles to networks with hundreds of different relations. We briefly demonstrate how statistical properties of structural presence make this feasible in time and space for most large, sparse networks using a worst-case random graph model. Finally, we select the incorporation of longitudinal information as a challenging problem in which vertex collocation profiles can offer new insights. We choose to view longitudinal data from a multirelational perspective and show that the incorporation of temporality in this way with vertex collocation profiles offers striking benefits in predictive efficacy.

All theory in the paper is distilled into clean, optimized implementations of asymptotically optimal algorithms in C++. The source code is freely and publicly available at https://github.com/rlichtenwalter/vcp. We have also incorporated VCP algorithms into the LPmade (Lichtenwalter and Chawla 2011) link prediction software freely and publicly available on MLOSS at http://mloss.org/software/view/307/.

### Organization

We first offer a new, more general formal definition of vertex collocation profiles in Section “Vertex collocation profiles”. We describe how graph isomorphism relates to our work, and we provide the relevant mathematics and theory to take the reader from a definition of graph isomorphism to a definition and mathematical implementation of vertex collocation profiles. In Section “Algorithms”, we move to a description of two simple and slow but general and easily understood algorithms. Then, we provide faster, more sophisticated algorithms and describe how and why they work. Having laid the foundation for understanding and implementing the techniques, we move to demonstrating that they are highly predictive, and Section “Data” introduces and describes the data sets employed to that effect. In Section “The VCP method and link prediction”, we demonstrate the impressive performance of VCP vectors in supervised link prediction. Finally, Section “VCPs and multirelational data” expands our empirical coverage by showing how the inherent multirelational nature of vertex collocation profiles is useful by successfully tackling the challenge of incorporating temporality in predictive models. We offer some concluding remarks in Section “Conclusions”.

## Vertex collocation profiles

**Figure 1:**
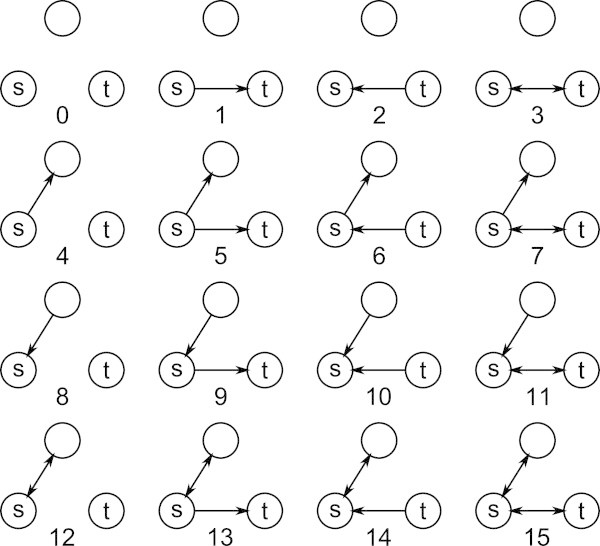
**Elements 0 through 15 of**  **.** Elements 16 through 63 are identical to their modulo 16 counterparts except for the presence of an out-edge, in-edge, or bidirectional edge connecting *v*_*t*_ to the free vertex.

Formally, a *vertex collocation profile* (VCP), written as , is a vector describing the relationship between two vertices, *v*_*i*_ and *v*_*j*_, in terms of their common membership in all possible subgraphs of *n*≥3 vertices over *r*≥1 relations, , with *d*∈{0=undirected,1=directed} directionality. A VCP *element*,  is defined as a distinct embedding of *v*_*i*_ and *v*_*j*_ within a particular isomorphism class of *n* vertices and is represented by a uniquely addressable cell in the VCP vector. Figure 1 illustrates the first 16 elements of . When referring to a VCP vector generally and not with respect to specific vertices, we can write VCP ^*n*,*r*,*d*^, excluding the subscript.

We can encode the connectivity in any multirelational network of *r* relations and *d* directionality with 2^(*d*+1)*r*^ different types of connections. Notably, we consider total lack of connectivity as itself a type of connection. Undirected unirelational networks exhibit two types of connectivity: existent and nonexistent. Directed unirelational networks exhibit four types of connectivity: nonexistent, outward, inward, and bidirectional. We choose not to consider self-loops in this treatment for clarity, but including them is theoretically and practically trivial.

The cardinality of VCP ^*n*,*r*,*d*^ depends upon the number of vertices, *n*, the number of types of relationship between them, , and whether or not directionality is considered, *d*. The space grows exponentially in the number of vertices with the base as the cardinality of the power set of undirected or directed relations. To explore this growth, we define a *subgraph universe* as the instantiation of G ^*n*,*r*,*d*^ with a specific (*n*,*r*,*d*) tuple so that the universe encompasses the collection of all possible subgraphs with *n* labeled vertices, *r* relations, and *d*∈{0=undirected,1=directed} directionality. Importantly, G ^*n*,*r*,*d*^ differs from VCP ^*n*,*r*,*d*^ in that the former has *n* labeled vertices and does not collapse isomorphically equivalent structures, whereas the latter has 2 labeled vertices and collapses isomorphisms on unlabeled vertices. It always holds that |*G*^*n*,*r*,*d*^|≥|*VCP*^*n*,*r*,*d*^|.

Equation 1 describes the number of subgraphs in *G*^*n*,*r*,*d*^. The base, 2^(*d*+1)*r*^, is the cardinality of the power set of edge types. The exponent, , describes the number of vertex pairs across which edges may be present.
1

Table 1 illustrates the number of subgraphs respecting vertex identity that compose a VCP given different values of *n* and *r* with *d*=0. This is a matrix of outputs from Equation 1.

**Table 1:** **Number of subgraphs composing VCP for values of** ***n*** **and** ***r*** **with** ***d*** **=0**

***r***	1	2	3	4
***n***				
3	8	64	512	4096
4	64	4096	262144	1.7×10^7^
5	1024	1048576	1.1×10^9^	1.1×10^12^
6	32768	1.1×10^9^	3.5×10^13^	1.2×10^18^

The number of subgraphs grows at a rate exponential in *n* and *r*, and incorporating directionality exacerbates the growth. The rate of growth of VCP vector cardinalities is much slower due to superlinear increases in the isomorphisms with increasing *n*, but VCP cardinality nonetheless grows quickly. Fortunately, the most important information is typically located close to the source and target vertices and is easily captured with small *n*, and many networks have only a few types of relationships.

### Isomorphisms

Isomorphic graphs are structurally equivalent graphs that vary in appearance only based on the identities of constituent vertices. More formally, we say that two multirelational graphs *G*=(*V*,*E*_1_,…,*E*_*r*_) and  are *isomorphic* if there exists a bijective function
2

and we can write *G*≃*G*^′^.

Isomorphism classes are sets of differing size containing all graphs within a given universe *G*^*n*,*r*,*d*^ that are isomorphic to a particular exemplar *G*∈*G*^*n*,*r*,*d*^. We can view this formally as
3

**Figure 2:**
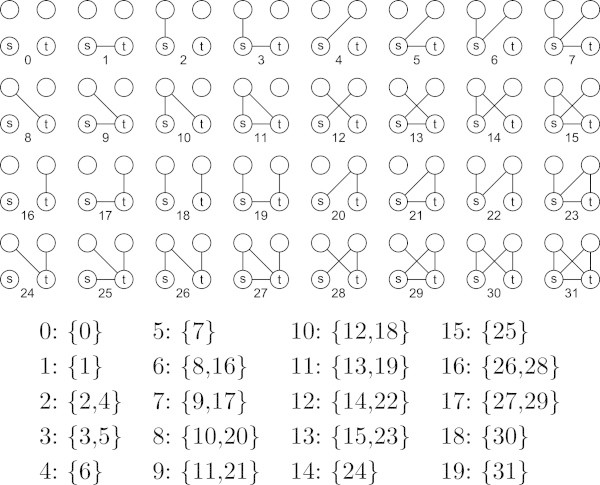
**Subgraphs 0 through 31 from**  **and the mapping of isomorphic subgraphs to VCP elements.** Structures 32 through 63 include a connection between the two free vertices and create 20 additional elements.

VCP elements are closely related to isomorphism classes. In Figure 2, it is impossible to distinguish subgraph 2 from subgraph 4. These subgraphs map to the same VCP element,  (2), and the count of that element is the sum of the counts of the isomorphic subgraphs.

Isomorphisms that require a mapping between *v*_*s*_ and *v*_*t*_, for instance subgraph 2 and subgraph 16 in Figure 2, do not share the same VCP element even though they reside within the same isomorphism class. VCP elements ignore isomorphisms that require mapping *v*_*s*_ to *v*_*t*_ because VCP describes the local embedding of these two explicitly identified vertices. In undirected graphs, elements such as  and  together supply information regarding symmetry or asymmetry in the density of the embedding of *v*_*s*_ and *v*_*t*_. The distinction in directed graphs is more obvious and relates to the potential significance of the difference in the local topologies of the source of a new link and its target. Figure 2 shows all the subgraphs pertinent to VCP ^4,1,0^ and their corresponding mappings to elements.

Thus we can say that , and we can say that two multirelational graphs *G* = (*V*,*E*_1_,…,*E*_*r*_) and  are *VCP-isomorphic* with respect to *s* and *t* if there exists a bijective function *f*_*s*,*t*_4

Determining the cardinality of VCP ^*n*,*r*,*d*^ is related to the complex problem of determining the number of isomorphism classes in a graph of *n* vertices. In VCP ^3,*r*,*d*^, each enumerated subgraph maps uniquely to a VCP element. *v*_*s*_ and *v*_*t*_ are not mappable, and there is only one permutation of the remaining vertex. In VCP ^4,*r*,0^, there are two mappable vertices and two permutations of those vertices. The number of mappable vertex permutations in *VCP*^*n*,*r*,*d*^ is described as (*n*−2)!, and the interaction of permutations with the appearance of isomorphisms is complex. The derivation of a general formula for |*VCP*^*n*,*r*,*d*^| for all *n*, *r*, and *d* is extremely combinatorially involved. We have instead provided software that computes VCP cardinalities and subgraph-to-element mappings for *n*∈ [ 3,*∞*), *r*∈ [ 1,*∞*), and *d*∈0,1. Table 2 shows the cardinality of all undirected VCPs with fewer than a million elements, but we shall demonstrate how it is possible in practice to perform analysis using VCP vectors with cardinalities many orders of magnitude greater than this.

**Table 2:** **Cardinality of**  **for values of** ***n*** **and** ***r*** **resulting in fewer than one million elements**

***r***	1	2	3	4	5
***n***					
3	8	64	512	4096	32768
4	40	2176	133120	-	-
5	240	183040	-	-	-
6	1992	-	-	-	-
7	24416	-	-	-	-

### Addressing

We define a VCP addressing scheme similar to the isomorphism certificate addressing scheme in (Kreher and Stinson 1999). The address space for the subgraphs from which the elements are derived is constructed by assigning *r* bits to each cell in the subgraph adjacency matrix and defining a significance value for the cell. The value of each edge in the matrix is defined as the index of the lexicographically ordered power set, , corresponding to the ordered set of  relations on that edge. Significance is assigned in increasing lexicographical order above the principal diagonal starting with *e*_1,2_ and ending with *e*_*n*−1,*n*_. Figures 1 and 2 demonstrate the indexing scheme for two different values of *n* and *r*. For any selection of vertices *v*_*s*=1_,*v*_*t*=2_,*v*_3_,…,*v*_*n*_, this addressing scheme will map the resulting multirelational subgraph to an index that exists within a set of indices of isomorphic structures. The procedure is defined and described more formally below.

Assume a square matrix  of order *n* representing adjacency of the following form with subscripts 5

in which cells indicate members of . In particular *a*_*i*,*j*_ corresponds to the binary-coded integral value of the bits denoting membership in the lexicographical ordering of .

Define a square matrix  of order *n* to represent the value of edges. This matrix will be used to provide a unique address for  through the grand sum of the Hadamard product of  and . For undirected networks, we define a matrix symmetric across the principal diagonal
6

to represent edges valued in increasing lexicographical order of their incident vertices. This matrix takes the following form, with each cell reserving *r* bits for the 2^*r*^ possible relations that may occur on the edge.
7

The symmetric property of  in the undirected case allows for symmetry in subgraphs with vertex permutations that cross the principal diagonal. For directed graphs, this symmetry is broken in vertex permutations and this must be reflected within the value matrix.
8

Each cell still reserves *r* bits to represent the 2^*r*^ values in , but an additional 2^*r*^ bits are reserved across the principal diagonal to represent  in the opposite direction giving the value matrix the following form.
9

Given the matrix  for multirelational adjacencies and the matrix  for edge values, we can compute the address *Ψ*(*G*_*x*_) of any subgraph *G*_*x*_∈*G*^*n*,*r*,*d*^ as the grand sum of their Hadamard product
10

This provides a complete addressing scheme for any number of subgraphs with any number of relations with or without directionality. To construct isomorphism classes within this addressing scheme, we start with a mapping vector
11

and execute all permutations of  from *i*=3 to *i*=*n*, which we define as the set . The exclusion of *i*=1 and *i*=2 from permutation excludes isomorphisms that result from mappings between *v*_*s*_ and *v*_*t*_. Each permutation corresponds to an isomorphic subgraph within the isomorphism class containing all of the permutations. To determine addresses for the permutations, we compute
12

We can define a *canonical* subgraph representative within an isomorphism class as
13

The set of canonical subgraph representatives as defined by the application of  to  in *G*^*n*,*r*,*d*^ is precisely the set of VCP ^*n*,*r*,*d*^ elements. The addresses for elements in VCP ^4,1,0^ are provided in Figure 2. Because manual identification of isomorphism classes is error-prone and difficult especially as the number of subgraphs increases, we have provided a program that outputs the mapping from all subgraph indices to their corresponding element addresses for all VCPs.

### Directionality

**Figure 3:**
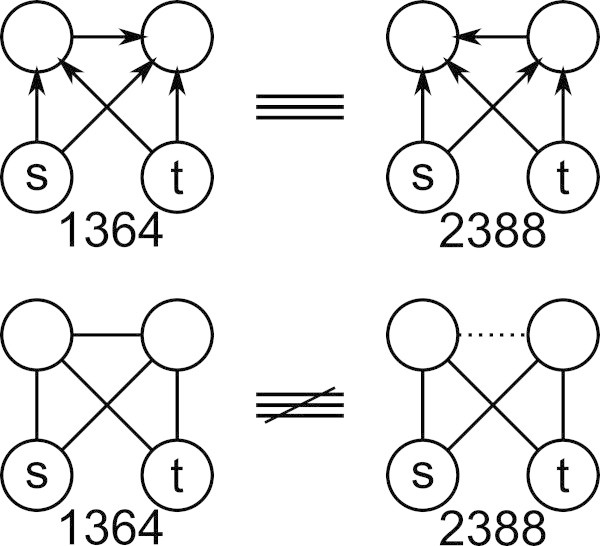
**Subgraphs and their addresses from VCP** ^**4,1,1**^ **(top) and VCP** ^**4,2,0**^ **(bottom).** The directed subgraphs (1364 and 2388) both map to VCP ^4,1,1^(884), but the multirelational subgraphs (1364 and 2388) map to VCP^4,2,0^(792) and VCP^4,2,0^(1336) respectively.

Directed networks with *r* relations are theoretically and practically distinct from undirected networks with 2*r* relations. The subgraph-to-element mapping differs with directed networks because  and  are no longer symmetric. This introduces more isomorphic equivalences and decreases the cardinality of a directed VCP vector by comparison to its undirected pseudoequivalent, a fact demonstrated in Figure 3. For instance, VCP ^4,1,1^, contains only 2112 elements whereas its undirected pseudoequivalent, VCP ^4,2,0^, contains 2176 elements.

### Dynamic vs. static computation

With matrices  and  and mapping vector , we have described the means by which one can generate the set of elements in an isomorphism class and the canonical representative of that class. We can enumerate the subgraph-to-element mapping by constructing the universe of  indexed in lexicographically increasing order, computing the canonical isomorphism class representative for each , and mapping the index to a contiguous, increasing value of VCP elements. This static computation determines VCP elements and addresses so that when a particular subgraph is identified, its VCP vector element may be determined with a constant-time operation in a precomputed vector.

As *n* and *r* increase, the number of subgraphs makes this static precomputation infeasible. We know from Equation 1 that enumerating members of *G*^*n*,*r*,*d*^ is an  operation. Determining the canonical representative of an arbitrary subgraph is an *O*(*n*!) operation, because , but all subgraphs discovered in the process no longer require the computation, so  necessarily bounds the total cost. Even so, with a complexity of , higher values of *n* and *r* make precomputation intractable. Even if computation were tractable, the size of the data structure may exceed memory and the size of the mapping may even exceed disk space.

Enumerating subgraphs and determining their isomorphism class is prohibitively expensive in many ways, but it is also unnecessary. We can instead dynamically compute the canonical representative of a given subgraph in *G*^*n*,*r*,*d*^ when we first observe it. Since we have not enumerated potential preceding VCP elements and since subgraph addresses of the canonical representatives may be extremely large as *n* and *r* grow, we cannot convert this canonical representation into a contiguous, invertible VCP element index and are thus forced to use an associative data structure with *O*(log*n*) rather than *O*(1) time lookup during counting. In losing this ability, however, we gain the ability to compute and represent VCP output in main memory so long as the population of the VCP vector is sufficiently sparse. In Section “Extension to complex networks”, we show that this sparsity requirement will almost always hold regardless of the (*n*,*r*,*d*) tuple.

## Algorithms

We have just described methods whereby the elements in a given VCP vector may be either enumerated or determined dynamically during counting. We now describe how to count the number of times each (*v*_*s*_,*v*_*t*_) pair appears embedded in a certain isomorphism class. We begin by introducing two algorithms that cover the simplest case where *n*=3. Then we cover algorithms for the much more complicated case where *n*=4. Several of the algorithms assume undirected graphs as input to simplify exposition, but all are easily generalizable to accept directed graphs.

For purposes of complexity analysis, we assume an underlying ordered compressed sparse row graph representation. This enables *O*(|*V*|) iteration through vertices and edge searches in *O*(log(|*Γ*(*v*_*i*_)|)) time, where *Γ*(*v*_*i*_) represents an ordered set of the neighbors of *v*_*i*_. The appearance of *Γ* in algorithms presents problems for complexity analysis, because the expectation of  is dependent on the kernel rather than just the first moment of the degree distribution of the graph (Jürgen and Carley 2012). Though the first moment of the degree distribution must always be  in either undirected or directed graphs, the distribution is often Poisson, log-normal, power law, or double Pareto log-normal (Reed and Jorgensen 2004), some of which possess expectations such that . The strict approach is to admit a worst-case distribution describing a star, correspondingly recognize that , and state |*Γ*(*v*_*i*_)|=*O*(|*V*|). We shall make the relaxed assumption, for more discriminative complexity analysis, that the distribution possesses a uniform kernel with expected value , and hence  implying . Then the approximation error in the case of an adversarial kernel is readily quantified as , reaching its maximum in sparse graphs when |*E*|≊|*V*| and its minimum in dense graphs when |*E*|≊|*V*|^2^.

Throughout the algorithms,  refers to a procedure to determine the index of the multirelational edge *e*_*i*,*j*_ in , the lexicographically ordered power set of relations. This procedure can derive power set indices efficiently by setting individual bits in the index according to the presence of the relation corresponding to that bit and indexing the bits by the natural order of the relations themselves. It is possible to maintain integers representing the power set index in the graph representation to allow accomplishing this in *O*(1) time.

Algorithm complexities are stated in terms of the time requirement for pairwise-independent VCP vector generation for a single pair of vertices. The edge search operation is implicitly indicated in the algorithms when *e*_*i*,*j*_ appears, and it refers to the query for an edge between *v*_*i*_ and *v*_*j*_.

### VCP^3,*r*,*d*^Algorithms

We begin by covering a member of the most naïve class of algorithms, represented by Algorithm 1. This algorithm is worth studying for three reasons. First, this algorithm is trivially generalized to any value of *n* by introducing an additional loop for each additional vertex and accumulating corresponding information about the new vertex pairings that become possible. Second, this algorithm most transparently describes the goal of the computation and is thus instructive for understanding the nature of VCP vectors. Third, for moderate values of *n*, the clarity of these algorithms allows for the production of test output with which to write more sophisticated algorithms.

#### Algorithm 1 VCP^3,*r*,1^(Naïve)



Though it offers conceptual advantages, this algorithm is unacceptably slow. Since it must loop through the set *V* to compute the vector, it requires *O*(|*V*|) edge searches. We can easily generalize this complexity for the entire class of naïve algorithms for any *n*. With each incrementation of *n*, we add an additional loop through the set *V*, yielding an *Ω*(|*V*|^*n*−2^) time, but we must also perform a number of operations in each inner loop related to the number of containing loops. The number of operations in the directed case and the asymptotic reduction in *n* and *V* applying also to the undirected case are written as
14

where the subscript *x* expresses each successively nested loop. The cost of the edge search operation has already been described as *O*(log|*Γ*(*v*_*i*_)|), and substituting the  approximation yields a general complexity for the naïve class of algorithms of . For Algorithm 1, the time complexity is .

Fortunately it is possible to do much better. We can recognize that, excluding the connection between *v*_*s*_ and *v*_*t*_ from consideration, connected structures must include members of *Γ*(*v*_*s*_) or *Γ*(*v*_*t*_). All structural counts are dictated by the cardinalities of the sets resulting from taking the differences and intersection of these. We can simultaneously account for unconnected structures by subtracting their union from *V*, excluding *v*_*s*_ and *v*_*t*_ from the result, and taking the cardinality of the remaining set. Algorithm 2 formalizes the undirected variant of the procedure.

#### Algorithm 2 **VCP**^**3,1,0**^



The analysis of this algorithm reduces to the analysis of the union, difference, and intersection set operations, all of which are easily implemented in *O*(|*U*|+|*T*|), where *U* and *T* are the respective sets. The algorithm also incurs the  cost of the search for *e*_*i*,*j*_, but this is subsumed when we substitute |*Γ*(*v*_*i*_)+*Γ*(*v*_*j*_)| for |*U*|+|*T*| and arrive at . In efficient implementations, the four set operations are collapsible into a single linear scan through *Γ*(*v*_*i*_) and *Γ*(*v*_*j*_), and such implementations allow the approach to handle any value of *r* through application of *Φ* to members of *Γ*(*v*_*i*_) and *Γ*(*v*_*j*_) with no change to asymptotic complexity.

The usage of set operations reduces the time requirement of the algorithm by a factor of . We shall next demonstrate that much greater savings are possible with *VCP*^4,*r*,*d*^ using more clever set operations and corrections for double-counting.

### VCP^4,*r*,*d*^Algorithms

The VCP ^3,*r*,*d*^ algorithms directly increment element counts corresponding to the underlying subgraph structure index, because no isomorphic equivalences are present with only one free vertex. In VCP ^4,*r*,*d*^ algorithms, the presence of two free vertices requires the determination of canonical isomorphism class representatives from observed structures. We designate this operation in the algorithms with *ε*, and implement it in *O*(1) time with a precomputed vector of mappings.

#### Algorithm 3 VCP ^4,*r*,0^(Naïve)



We first provide Algorithm 3 as an undirected exemplar of the class of naïve algorithms applicable in the case of four vertices. It works by iterating through every pair of free vertices in the graph and considering them in combination with *v*_*s*_ and *v*_*t*_ to count the resulting structures. As demonstrated by the generalized complexity analysis provided above, its asymptotic complexity is .

It is again possible to reduce the complexity by restricting consideration to neighbors, but the procedure is more complex. The algorithm commences by considering the neighbor sets of *v*_*s*_ and *v*_*t*_ to compose structures of three vertices. These structures are extensible either by the addition of another neighbor of either *v*_*s*_ or *v*_*t*_ or by the addition of a neighbor of their neighbors. To calculate the counts of sparse structures, which are too numerous to cheaply enumerate, the algorithm maintains counts of the number of observed connected pairs, the number of observed unconnected pairs, and the number of vertices that are two hops from *v*_*i*_ or *v*_*j*_. They are written as *α*, *β*, and *γ* respectively. These quantities provide sufficient information to differentiate unobserved structures in which the two free vertices are isolates within the subgraph from those in which they are isolated from *v*_*s*_ and *v*_*t*_ but connected to each other. Algorithm 4 formalizes the procedure and provides more details.

#### Algorithm 4 **VCP**^**4,1,0**^



All set operations require  time. Considering the  neighbors for the outer loop (line 4) requires  time. Inside the loop, the algorithm performs edge searches requiring  time and performs additional set operations requiring  time. The inner loops (line 19 and line 25) both involve  iterations, and incur  cost for edge searches. We can express the critical components of the cost mathematically as
15

demonstrating that graph sparsity is an important factor in the feasibility of the algorithm. **VCP**^**4,1,0**^

We call special attention to three lines in Algorithm 4 the purposes of which may not be obvious. Line 37 computes the number of structures in which the *v*_*k*_ under consideration is a member and in which a fourth vertex is isolated and thus unexplored through consideration of neighbors. This is equivalently the number of vertices in the network that are neither *v*_*i*_ nor *v*_*j*_ nor a neighbor of *v*_*i*_, *v*_*j*_, or *v*_*k*_, and it is given by the expression .

Lines 39 and 40 consider structures in which *v*_*k*_ and *v*_*l*_ are connected to neither *v*_*i*_ nor *v*_*j*_. Such structures are divided into two cases: one in which *v*_*k*_ and *v*_*l*_ are connected to each other and one in which they are not. Counting these structures directly is expensive, because *v*_*k*_ and *v*_*l*_ are not explored as neighbors of *v*_*i*_ and *v*_*j*_. Fortunately careful curation of the number of previously encountered connections and gaps facilitates the computation of these quantities. Line 40 computes the number of structures where *v*_*k*_ and *v*_*l*_ are connected, which is equivalent to the total number of edges in the network excepting those encountered directly during counting. Line 40, which computes the number of structures where *v*_*k*_ and *v*_*l*_ are not connected to each other, is significantly more complicated to derive. We start with the number of gaps we know to exist in the network, . From this, we must subtract all of the gaps that we encountered while counting, expressed as *β*, but we must also remember that *β* only describes the number of gaps directly observed. We must additionally incorporate gaps contributed by the structure count computed by line 37.

Recall that |*V*−(*Γ*(*v*_*i*_)∪*Γ*(*v*_*j*_)∪*Γ*(*v*_*k*_)∪{*v*_*i*_,*v*_*j*_})| is the number of such structures. Outside the loop, this number may be expressed in aggregate by the product of the number of vertices that may serve as *v*_*k*_, written as , and the number of vertices that are never *v*_*i*_, *v*_*j*_, *v*_*k*_, or *v*_*l*_, written as . This expression gives the number of such structures, but this only accounts for gaps not directly observed between *v*_*k*_ and *v*_*l*_. The sparse subgraphs also contribute unobserved gaps involving *v*_*i*_ and *v*_*j*_, and we can express these with . Subtracting these two quantities from the number of possible gaps minus the number of directly observed gaps provides us with the count of the least connected isomorphism class present in . This explication also applies to Algorithm 5, which has identical computations at lines 36, 38, and 39.

We can further improve Algorithm 4 by recognizing that the edge searches are redundant. Since the algorithm encounters all vertices connected to *v*_*s*_ and *v*_*t*_ during the set operations, it can maintain knowledge of pertinent edges to record structures while performing those operations. The procedure is described in Algorithm 5. In fact, it is possible to implement the algorithm with only two outer loops through *Γ*(*v*_*i*_)∪*Γ*(*v*_*j*_) in which one of those loops contains a simultaneous nested loop through *Γ*(*v*_*i*_)∪*Γ*(*v*_*j*_) and *Γ*(*v*_*k*_) where *v*_*k*_ is the current neighbor considered by the outer loop. An efficient implementation consists almost entirely of additions, dereferences, and branches and imposes only a moderate constant factor on the asymptotic complexity. By removing the  contributions of those searches from the summations in Equation 15, we reduce the asymptotic complexity of the algorithm to . This complexity applies also to directed and multirelational graphs and is asymptotically optimal for VCP ^4,*r*,*d*^ in the regime of pairwise-independent computation. Compared to the naïve algorithm for four vertices, the time complexity is reduced by a factor of .

#### **Theorem1**

Given a graph *G*=(*V*,*E*) with uniform degree distribution and vertices *v*_*i*_ and *v*_*j*_, pairwise-independent computation of  in *G* has complexity .

#### **Proof1**

We observe that  describes the number of pairs comprising vertices that are both unreachable from *v*_*i*_ and *v*_*j*_ within geodesic distance *ℓ*. Because the quantities are combinatorially related, it also describes the number of vertices individually unreachable from *v*_*i*_ and *v*_*j*_ within *ℓ*. Determining this quantity requires at least as much work as determining the number of vertices unreachable only from *v*_*i*_ within *ℓ*. Since VCP ^4,*r*,*d*^ allows two free vertices, *ℓ*=2. The fastest way to determine reachability in the worst case is employing a breadth-first search rooted at *v*_*i*_, requiring  time. Since VCP ^4,*r*,*d*^ computation determines the reachability of vertices from *v*_*i*_, it must share the lower bound and is .

#### **Corollary1**

Given a graph *G*=(*V*,*E*) with uniform degree distribution and vertices *v*_*i*_ and *v*_*j*_, pairwise-independent computation of  in *G* has complexity .

We summarize the algorithms and complexities covered in Table 3. The final row does not reflect a specific provided algorithm but instead addresses the general theoretical bound on how well any pairwise-independent VCP computation may perform.

**Table 3:** **VCP algorithms and their complexities**

Algorithm	Complexity	Pairwise-independent
		optimality
VCP ^3,*r*,*d*^ (Naïve)		
VCP ^3,*r*,*d*^		✓
VCP ^4,*r*,*d*^ (Naïve)		
VCP ^4,*r*,*d*^ (Improved)		
VCP ^4,*r*,*d*^		✓
VCP ^*n*,*r*,*d*^		N/A

### Extension to complex networks

It is mostly trivial to extend VCP algorithms to more complex networks, including any form of edge feature such as directionality, weight, temporality, different relation types, or any information describing edges or vertex pairs that either exists categorically or can be quantized. One amenable network representation associates an ordered set of bits with each edge. Each bit corresponds to the presence of a particular relation or some Boolean descriptor for a pair of vertices. The determination of the existence of an edge for unirelational data instead becomes an evaluation of the edge as the binary-coded integral value of the ordered set of bits. This is one conceivable implementation for  in Algorithm 1.

A complication emerges as *r* increases related to determining counts of multirelational edges in subgraphs with disconnected substructures. VCP ^*n*,*r*,*d*^ counts structures including those in which none of 1≤*k*≤*n*−2 vertices *v*_*i*_,*v*_*i*+1_,…,*v*_*i*+*k*−1_∈{*v*_3_,*v*_4_,…,*v*_*n*_} may be connected to either *v*_1_ or *v*_2_. It is impossible to take advantage of set operations on sets of neighbors because *v*_*i*_ is not in a neighbor set. There are two alternative methods of counting. The first, computationally infeasible even for small networks, is to enumerate all  sets of vertices. The second approach, which works well for *n*=4 and generalizes for *n*>4, is to associate with each existing member of  the count of its occurrence and decrement the count each time a corresponding member is encountered in computing a given vector . Knowing how many of the  connectivities the graph contains and how many are present in direct involvement with *v*_*i*_ and *v*_*j*_, it is possible to determine the count of connectivities of vertices not in direct involvement. This admits a correct count of VCP elements. While this method will outperform enumeration, it can still become problematic when *r* is large because of the space and time involved in creating and maintaining the counts. Though we included these structures in our counts, empirical results show that surveying the connectivity of subgraphs with no direct connectivity to *v*_*i*_ and *v*_*j*_ reveals much less information than surveying connectivity within subgraphs connected to *v*_*i*_ and *v*_*j*_. Omitting this information from the VCP vector offers an opportunity for increases in speed.

The feasibility of multirelational applications of VCP analysis with *r*≫10 depends a lot upon the number of VCP elements that are populated. Even before the number of enumerable subgraphs reaches values such as 2^60^ for *G*^4,10,0^, storing the complete VCP vector becomes impossible. Fortunately, most graphs are friendly to VCP analysis, and only an infinitesimal fraction of the total number of possible isomorphism classes is present. Theoretical statements regarding expectations for the number of VCP elements in various random graph models may be possible, but we turn to strong empirical demonstrations corresponding to worst-case scenarios. In the most difficult case, VCP analysis must describe all pairs of vertices in a graph with moderate density and random linkage. Coverage of all pairs guarantees that any existing structures must be observed. Moderate density is necessary to create a large population of both sparsely and densely connected subgraphs. Random linkage is necessary to ensure that the sample exhibits a variety of isomorphism classes.

**Figure 4:**
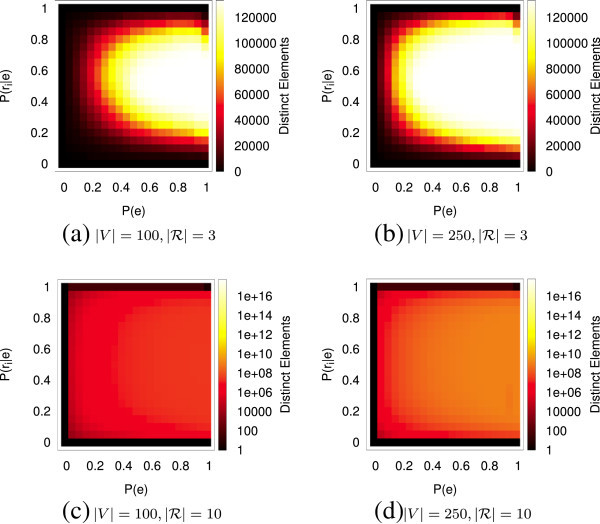
**All-pairs VCP element population for**  **.** Note the logarithmic heatmap scale on the bottom two  plots, which is necessary to prevent the plots from appearing solid black due to extreme element sparsity. **(a)** , **(b)** |*V*|=250, , **(c)** , **(d)** .

We simulate this adversarial environment with a specialized Erdös-Renyi random graph model with four parameters: . Since we will perform VCP analysis on all pairs, |*V*| places the effective upper bound on the number of different structures we can observe, . The value  is the number of different relations that can exist over an edge, and it controls the cardinality of the set of isomorphism classes. The probabilistic parameter *P*(*e*_*i*,*j*_) is the traditional *p* parameter from the *G*(*n*,*p*)-model Erdös-Renyi random graph except that it controls whether or not an edge is a candidate for multirelational population. For edges whose assigned uniform random value falls below *P*(*e*_*i*,*j*_), the probabilistic parameter  is used as a threshold against which uniform random values are tested for each of the *r* possible relations to determine the relations that will apply on that edge. Figure 4 shows the number of VCP elements observed in all-pairs analysis of 1,764 random graphs constructed in this manner.

For densities below 30%, there are simply too few edges to allow for the formation of dense isomorphism class exemplars, and less than 50% of the possible VCP ^4,*r*,0^ elements appear. The probability of observing relations on edges is also influential.  must be neither extremely low, in which case all substructures are sparse, nor extremely high, in which case all substructures are fully populated with all relations. The lower the density, the more critical it is that the relational probabilistic parameter be in a moderate zone. Extremely high density alone is not sufficient to prevent the formation of most isomorphism classes since *P*(*e*_*i*,*j*_)=1 does not necessarily imply a link and because the random selection of elements from  will still allow for the construction of many isomorphism class exemplars even when involving every edge in the network.

The graphs with |*V*|=250 offer  selections of 4 vertices whereas the |*V*|=100 offer only  selections of 4 vertices. The heat maps indicate that observed diversity is limited by random chance of appearance when the number of subgraphs increases or the number of possible selections of vertices decreases. This manifests as a decrease in the size of the region where many elements are observed in terms of the two probabilistic parameters and introduces another helpful mechanism in maintaining manageable VCP element presence.

In reality, the situation is much less adversarial and more conducive to VCP analysis than this. First, as *r* increases to extreme values and |*VCP*^*n*,*r*,*d*^| increases as a result, the exponential growth in the cardinality of the set of isomorphism classes quickly outstrips , which places an effective upper bound on the number of possible elements. More significantly, densities are often fractional percentages of their maximum values, so typical analytical tasks operate in areas of the heat map even more favorable than those displayed. It is also rare for all relations to be highly and equally common across the entire network. Links usually form according to underlying guiding motivations, which induce structural similarities throughout the topology. Taken together, these greatly limit the size of the observed subset of highly multirelational VCP vectors to manageable output sizes. Further, as we shall show in Section “Computational challenges”, these factors also work to limit the entropy of the values within any particular VCP element.

## Data

We present results for several data sets in Table 4 to demonstrate the performance of the techniques under comparison for different families of networks. Though all of these data sets contain information with which to generate edge weights, we are interested in providing purely structural comparison here, so all quantitative results are based on networks constructed without edge weights.

**Table 4:** **Some basic properties of the data sets**

Name	Directed	Vertices	Edges		***r***_***a***_
calls	✓	7,786,471	33,292,508	0.127	0.212
condmat		17,216	110,544	0.642	0.177
dblp-cite	✓	15,963	344,373	0.128	-0.046
dblp-collab		367,725	2,088,710	0.617	0.254
disease-g		399	15,634	0.665	-0.310
disease-p		437	81,158	0.818	-0.406
hepth-cite	✓	8,249	335,028	0.352	0.097
hepth-collab		8,381	40,736	0.466	0.237
huddle		4,243	997,008	0.591	-0.211
patents-collab		1,162,227	5,448,168	0.531	0.141
sms	✓	5,016,746	11,598,843	0.048	0.042

These figures are reported for networks constructed using all available longitudinal data.  represents average clustering coefficient and *r*_*a*_ represents assortativity coefficient.

calls is a stream of 262 million cellular phone calls from a major cellular phone service provider. We construct directed networks from the calls by creating a node *v*_*i*_ for each caller and a directed link *e*_*i*,*j*_ from *v*_*i*_ to *v*_*j*_ if and only if *v*_*i*_ calls *v*_*j*_. To reduce the noising effect of robot callers and automated answering systems, we exclude a few nodes that only call if they have an out-degree above 1000 or only receive calls if they have an in-degree above 1000. sms is a stream of 84 million text messages from the same source as calls and constructed in the same manner.

condmat (Newman 2001a) is a stream of 19,464 multi-agent events representing condensed matter physics collaborations from 1995 to 2000. We construct undirected networks from the collaborations by creating a node for each author in the event and an undirected link connecting each pair of authors. For all experiments involving condmat, we use the years 1995 to 1999 for constructing training data and the year 2000 for testing.

dblp-cite (Ley 2002) is a citation network based on the DBLP computer science bibliography. Each researcher is a node *v*_*i*_ and directed networks are formed by viewing a citation by researcher *v*_*i*_ of work by researcher *v*_*j*_ as a directed link *e*_*i*,*j*_. The dblp-collab network uses the same raw data, but links are based on co-authorship collaborations. An undirected link exists between *v*_*i*_ and *v*_*j*_ if both are authors on the same paper.

disease-g (Davis and Chawla 2011) is a network in which nodes represent diseases and the links between diseases represent the co-occurrence of particular genotypic characteristics. Links are undirected. This network is not longitudinal, but finding unobserved links is an important task, so we have no choice but to estimate performance by randomly removing links to construct test sets. The disease-p network is from the same source as disease-g, but links in disease-p represent the co-occurrence of phenotypic characteristics. Predictions of common expressions between diseases are uninteresting since expressions are either observed between diseases or they are not. Because of this, practically speaking the value of phenotypic predictions is negligible. Nonetheless, holding out phenotypic links and subsequently predicting their presence is equally instructive for the purposes of predictor evaluation.

hepth-cite and hepth-collab (McGovern et al. 2003) are formed in exactly the same way as dblp-cite and dblp-collab respectively. The raw data for these networks is a set of publications in theoretical high-energy physics.

The huddle data set from (Raeder et al. 2011) is transaction data gathered at a convenience store on the University of Notre Dame campus. The data was collected from June 2004 to February 2007. Products are represented by nodes, and products purchased together in the same transaction are represented by undirected links.

The patents-collab (Valverde et al. 2007) data set is constructed from the data at the National Bureau of Economic Research. Nodes represent authors of patents and undirected links are formed between authors who work together on the same patent.

## Computational feasibility

To demonstrate computational feasibility, we computed VCP vectors serially on one core with commodity desktop hardware. Our machine included an Intel Core i5-2500K running at 4.8 GHz with 6 MB of level 3 cache and 8 GB of 1600 MHz dual-channel memory. Table 5 shows the results of computing the vectors for all *ℓ*=2 pairs, pairs spanning a geodesic distance of 2. Despite the positive serial feasibility, VCP algorithms are naturally parallel: each vector computation is completely independent of the others, so multi-core, grid, or cloud distribution allows for perfect linear speedup. Despite the 8 GB of memory available on the machine, we note that, aside from the graph representations themselves, actual processing of all algorithms required less than 128 KB for all data sets and fit into the 32 KB level 1 cache for most. Nevertheless, we also developed and make available *O*(1) space algorithms that are only slightly slower.

**Table 5:** **Network statistics (a), wall clock running times (b), and throughput (c) pertinent to scalability of VCP algorithms**

(a) Network statistics				
**Name**	**Vertices**	**Edges**	**Density**	***ℓ*** **=2 Pairs**
calls	7,786,471	33,292,508	5.49×10^−7^	2.46×10^8^
condmat	17,216	110,544	7.46×10^−4^	3.80×10^5^
dblp-cite	15,963	344,373	1.35×10^−3^	1.97×10^7^
dblp-collab	367,725	2,088,710	3.09×10^−5^	1.07×10^7^
disease-g	399	15,634	1.97×10^−1^	5.87×10^4^
disease-p	437	81,158	8.52×10^−1^	5.46×10^4^
hepth-cite	8,249	335,028	4.92×10^−3^	1.23×10^7^
hepth-collab	8,381	40,736	1.16×10^−3^	1.45×10^5^
huddle	4,243	997,008	1.11×10^−1^	7.29×10^6^
patents-collab	1,162,227	5,448,168	8.07×10^−6^	2.50×10^7^
sms	5,016,746	11,598,843	4.61×10^−7^	4.74×10^7^
**(b) Wall clock running times (in seconds)**				
**Name**	**VCP** ^**3,1,0**^	**VCP** ^**3,1,1**^	**VCP** ^**4,1,0**^	**VCP** ^**4,1,1**^
calls	85.0	531.2	2660.0	3763.0
condmat	0.1	-	2.6	-
dblp-cite	23.9	120.8	11561.7	19686.6
dblp-collab	2.7	-	149.3	-
disease-g	0.1	-	2.3	-
disease-p	0.1	-	13.9	-
hepth-cite	25.0	125.4	12830.1	24818.7
hepth-collab	0.1	-	0.8	-
huddle	17.4	-	14360.3	-
patents-collab	7.7	-	462.3	-
sms	10.7	66.1	177.6	242.5
**(c) Throughput (pairs per second)**				
**Name**	**VCP** ^**3,1,0**^	**VCP** ^**3,1,1**^	**VCP** ^**4,1,0**^	**VCP** ^**4,1,1**^
calls	2.9×10^6^	4.6×10^5^	9.3×10^4^	6.5×10^4^
condmat	5.0×10^6^	-	1.4×10^5^	-
dblp-cite	8.2×10^5^	1.6×10^5^	1.7×10^3^	1.0×10^3^
dblp-collab	3.9×10^6^	-	7.1×10^4^	-
disease-g	2.0×10^6^	-	2.6×10^4^	-
disease-p	6.7×10^5^	-	3.9×10^3^	-
hepth-cite	4.9×10^5^	9.9×10^4^	9.6×10^2^	5.0×10^2^
hepth-collab	4.8×10^6^	-	1.8×10^5^	-
huddle	4.2×10^5^	-	5.1×10^2^	-
patents-collab	3.3×10^6^	-	5.3×10^4^	-
sms	4.4×10^6^	7.2×10^5^	2.6×10^5^	1.9×10^5^

Density is reported as  in undirected networks and  in directed networks. Stated running times and throughputs are the arithmetic mean of 10 runs, but variances are negligible.

## The VCP method and link prediction

Link prediction has been defined and framed in many ways, but it is in essence the task of accepting a network *G*=(*V*,*E*) and predicting whether there is or will be an unobserved link *e*_*s*,*t*_ between a pair of nodes *v*_*s*_ and *v*_*t*_ where *v*_*s*_,*v*_*t*_∈*V* and *e*_*s*,*t*_∉*E*. In multirelational networks, we simply accept that *E* is a vector, and we often also interested in predicting both the relational type and existence of a link. VCPs offer several advantages in tackling link prediction. These include incorporating many different hypotheses of underlying link formation mechanisms through the identification of all vertex relationships in subgraphs of size *n* and natively handling multirelational data.

We compare the link prediction efficacy of VCPs with that of a selection of other methods. We select representatives from different predictor families established as strong by prevailing literature (Liben-Nowell and Kleinberg 2007). Unsupervised methods include the Adamic/Adar predictor based on common neighbors (Adamic and Adar 2001), the Katz path-based predictor (Katz 1953), and the preferential attachment model (Barabási et al. 2002; Newman 2001b). We also compare against the HPLP supervised link prediction framework contributed by (Lichtenwalter et al. 2010), which combines a variety of unsupervised predictors into a classification model.

### Experimental setup

To run our experiments, we integrated VCP with the LPmade link prediction software (Lichtenwalter and Chawla 2011). LPmade uses a GNU make architecture to automate the steps necessary to perform supervised link prediction. This integration ensured a fair and comparable testing regime across predictors and allows those interested in VCP for link prediction and other purposes to test it on their networks easily.

To perform supervised classification with a VCP vector, we use the unmodified VCP vector, , as the classification feature vector. The class label is then determined by the existence or nonexistence of the edge (*v*_*i*_,*v*_*j*_) in a future form of the network. Non-longitudinal data is handled in the same way as longitudinal data by randomly assigning timestamps to edges, so for non-longitudinal data the future form of the network corresponds to a random hold-out of edges. Classification data is not divided from a single data set generated from a single underlying network but is instead constructed from different underlying network data. Training data uses older forms of the network than testing data, and training labels always come from data strictly preceding the testing label period to prevent data leakage. Experiments are fully reproducible through the use of the LPmade framework.

When performing classification using VCPs, we opted for the bagged (Breiman 1996) random subspaces (Ho 1998) implementation from WEKA (Witten and Frank 2005). This classification scheme offers lower peak memory requirements than random forests while simultaneously providing the potential to handle feature redundancy (Ho 1998). We considered presenting results with HPLP also using random subspaces, but we determined that random subspaces produced decreased or comparable performance to the original reference implementation, so we present HPLP results unmodified using random forests (Breiman 2001).

We used the default values from HPLP of 10 bags of 10 random forest trees, 10 bags of 10 random subspaces for VCP classifiers, and training set undersampling to 25% positive class prevalence. We did not change the size or distribution of the testing data. For undirected networks, we resolve *f*(*v*_*s*_,*v*_*t*_)≠*f*(*v*_*t*_,*v*_*s*_), by computing the arithmetic mean to serve as the final prediction output. By default, LPmade includes features that consider edge weights such as the sum of incoming and outgoing link weights. We are interested in the comparative prediction performance of the link structure alone, so we ran all predictors on the networks disregarding edge weights. There are many different ways to assign edge weights to all the networks here, and the particular choice of edge weight and the precise decision about how to incorporate it into the VCPs would distract from the study.

Computing and evaluating predictions using any prediction method for all possible links on large, sparse networks is infeasible for multiple computational reasons including time and storage capacity. Link sparsity is likewise challenging for statistical modeling because of extremely low prior probability of link formation (Getoor 2003; Lichtenwalter et al. 2010; O’Madadhain et al. 2005; Rattigan and Jensen 2005). Link prediction within a two-hop geodesic distance provides much greater baseline precision in many networks (Lichtenwalter et al. 2010; Scellato et al. 2011), so effectively predicting links within this set offers a strong indicator of reasonable deployment performance. For all compared prediction methods, we restricted the prediction task by distance and only considered performance comparisons for potential links spanning two hops within the training data due to their higher prior probability of formation and computational feasibility.

### Prediction performance

The area under the receiver operating characteristic curve (AUROC) can be deceptive in scenarios with extreme imbalance (Hand 2009), and area under the precision-recall curve (AUPR) exhibits higher sensitivity in the same scenarios (Davis and Goadrich 2006). We will provide results for those interested in traditional AUROC, but we will also present AUPR results and will mainly restrict our analysis to those results. Table 6 shows the comparative AUROC and AUPR performance of Adamic/Adar, Katz, preferential attachment, HPLP, and VCPs in link prediction for potential links spanning a geodesic distance of two hops.

**Table 6:** **Comparative AUROC (top) and AUPR (bottom) performance for Adamic/Adar (AA), Katz, preferential attachment (PA), HPLP, and VCP**

(a) AUROC								
	**AA**	**Katz**	**PA**	**HPLP**	**VCP** ^**3,1,0**^	**VCP** ^**3,1,1**^	**VCP** ^**4,1,0**^	**VCP** ^**4,1,1**^
calls	0.698	0.641	0.424	0.782	0.802	0.814	0.834	**0.849**
condmat	**0.663**	0.630	0.585	0.588	0.637	-	0.582	-
dblp-cite	0.794	0.791	0.773	0.841	0.830	0.847	0.843	**0.868**
dblp-collab	**0.697**	0.623	0.523	0.691	0.640	-	0.695	-
disease-g	0.930	0.907	0.820	**0.970**	0.923	-	0.964	-
disease-p	0.898	0.920	0.932	0.922	0.939	-	**0.951**	-
hepth-cite	0.826	0.794	0.766	0.838	0.836	0.846	0.845	**0.851**
hepth-collab	0.606	0.619	0.547	0.592	0.598	-	**0.622**	-
huddle	0.879	0.875	0.875	0.877	0.881	-	**0.888**	-
patents-collab	0.793	0.671	0.532	0.800	0.680	-	**0.816**	-
sms	0.642	0.581	0.472	0.714	0.735	0.730	0.791	**0.802**
**(b) AUPR**								
	**AA**	**Katz**	**PA**	**HPLP**	**VCP** ^**3,1,0**^	**VCP** ^**3,1,1**^	**VCP** ^**4,1,0**^	**VCP** ^**4,1,1**^
calls	0.000505	0.011465	0.000092	0.018005	0.031655	0.033091	0.033533	**0.035127**
condmat	0.000195	0.000183	0.000177	0.007763	**0.011917**	-	0.008589	-
dblp-cite	0.000314	0.000246	0.000234	0.016030	0.009207	0.015265	0.011427	**0.018137**
dblp-collab	0.008777	0.006723	0.003251	0.007772	0.007152	-	**0.009410**	-
disease-g	0.221299	0.193863	0.061694	**0.466716**	0.155165	-	0.444153	-
disease-p	0.629516	**0.676419**	0.673601	0.390074	0.552765	-	0.633316	-
hepth-cite	0.003967	0.003784	0.003225	0.054846	0.046140	0.059245	0.056244	**0.063387**
hepth-collab	0.008563	**0.009328**	0.005060	0.006123	0.007197	-	0.007156	-
huddle	0.000790	0.000746	0.000745	0.039914	0.039394	-	**0.046803**	-
patents-collab	0.006962	0.005678	0.001684	0.006735	0.005564	-	**0.007709**	-
sms	0.009410	0.009164	0.002986	0.011594	0.025206	0.026063	0.027073	**0.028201**

Bold font indicates the maximum value in the row.

In general, we expect the information content of VCPs to increase in the left-to-right order presented in Table 6, though depending on the significance of directedness in the network, the expectation of comparative performance of VCP ^3,1,1^ and VCP ^4,1,0^ may change. We point the reader to calls, dblp-cite, dblp-collab, disease-g, disease-p, hepth-cite, huddle, patents-collab, and sms as conformant examples. We suspect that the exceptions indicate cases in which the classification ensemble failed to create a sufficiently optimized model in the high-dimensional space.

In 7 of the 11 networks, VCP classification offers superior AUPR performance. In a slightly different selection of 7 networks, it offers superior AUROC performance. In some of the cases in which VCP offers the best performance, the differences are quite wide. In the sms network it offers an AUPR that is 2.3 times as high as the best competitor. In the condmat network, it offers AUPR 1.53 times the nearest competitor. In two of the networks in which VCP classification does not provide the best performance, HPLP does. As an interesting side note, when weights are removed as they were to obtain these results, HPLP does not always outperform the unsupervised predictors.

**Figure 5:**
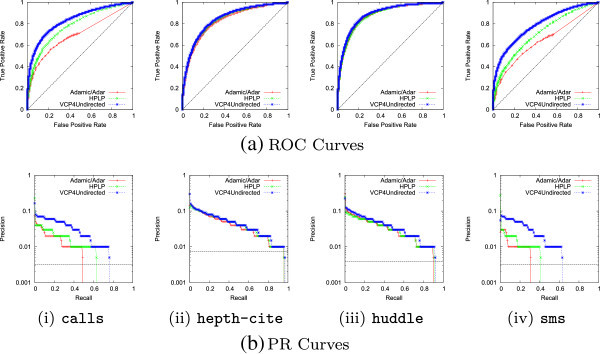
**ROC (top) and precision-recall (bottom) curves for selected networks.** **(a)** ROC Curves, **(b)** PR Curves: (i) calls, (ii) hepth-cite, (iii) huddle, (iv) sms.

Figure 5 shows a closer look at the performance differences. The black dashed line represents the baseline performance of a random predictor. Across all the selected networks, VCP maintains high precision longer at increasing values of recall. This is especially important in link prediction where high precisions are so difficult to achieve.

Despite the competitive performance that the VCP method exhibits, it is not our intent to present the most effective possible classification scheme. There is great potential for improvement through feature selection, dimensionality, and alternative classification algorithms. Increasing the number of trees in the forest is also likely to improve performance in the complex feature space. Yet another option for potential improvement is to concatenate VCP ^3,*r*,*d*^ and VCP ^4,*r*,*d*^ into a single feature vector. VCP vectors contain a wealth of information, and the task is simply to determine how best to employ it to achieve whatever goals are desired.

## VCPs and multirelational data

**Figure 6:**
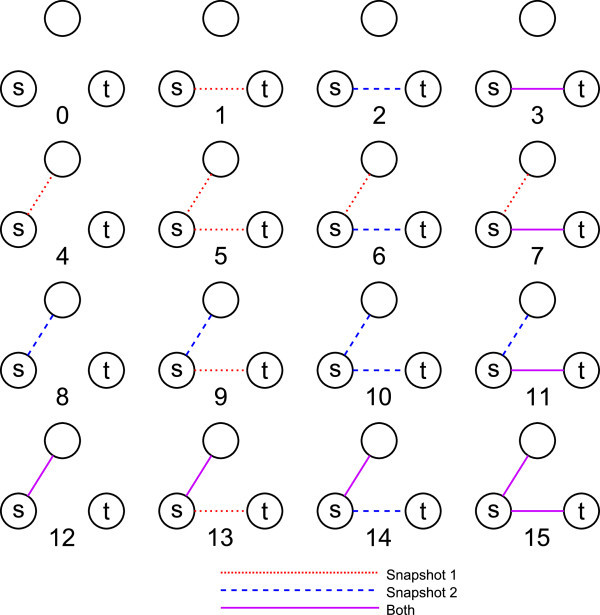
**Elements 0 through 15 of VCP** ^**3,2**^ **.** Elements 16 through 63 are identical to their modulo 16 counterparts except for the presence of various relations between *v*_*t*_ and the free vertex in one or more of the time periods.

In VCP algorithms, any procedures that consider a particular edge for unirelational data can instead determine the value of the edge as the binary-coded integral value of the ordered set of bits for multirelational data. Excepting the cost of allocating storage necessary to maintain counts for multirelational structural elements, the computational complexity of the multirelational extension is no greater than for unirelational networks. Figure 6 shows the first 16 elements of VCP ^3,2,0^.

We can capture the power of longitudinal data by encoding the snapshot in which an event occurs as its relation type. Consistent with the approach proposed by (Sharan and Neville 2008) and subsequently used by (Acar et al. 2009), we also divide our data into a set of discrete, non-overlapping snapshots. Suppose a continuous stream of event records arrives with arbitrarily fine temporal granularity. We divide the stream into *t*+1 chunks *s*_0_ to *s*_*t*_ sized, electively equally, according to selected criteria. In some cases, data is generated by a non-stationary process with periodicity, and so encapsulating the periodicity consistently might be an important criterion. From the chunks, we can create corresponding graph snapshots *G*_0_ to *G*_*t*_ such that all events involving entities *i* or *j* in arbitrary chunk *s*_*a*_ appear as links *e*_*i*,*j*_ between vertices *v*_*i*_ and *v*_*j*_ in *G*_*a*_. When we want a graph snapshot to be more temporally expansive than a single chunk, say including *s*_*a*_,*s*_*a*+1_,…,*s*_*a*+*b*_ with 1<*b*≤*t*−*a*, we can either compute the minimum common supergraph of multiple consecutive graph snapshots or equivalently compute a new graph snapshot containing the events within the desired bounds.

**Figure 7:**
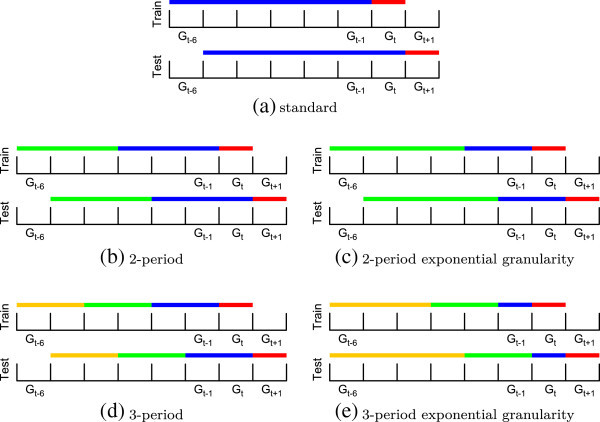
**Supervised classification approaches involving different temporal snapshot distributions.** Supervised classification approaches involving different temporal snapshot distributions. Different colors within the training data or testing data represent distinct snapshots. **(a)** standard, **(b)** 2-period, **(c)** 2-period exponential granularity, **(d)** 3-period, **(e)** 3-period exponential granularity.

When performing supervised learning on longitudinal data, conceptual and practical complexities arise that are absent from standard supervised learning tasks. These complexities are covered in great detail in the expansive literature on data stream mining (Gaber et al. 2005), and many of the topics endemic to all mining tasks on longitudinal data apply here. Figure 7 demonstrates the specific methodology employed within our experiments. As with non-longitudinal classification problems, there exists a model construction step using training data and a performance evaluation or deployment prediction step using testing data. The basic application of supervised learning to link prediction on longitudinal data is depicted in Figure 7a as a reference. Each color in each of the training and testing time lines represents a separate network snapshot.

In the basic application in Figure 7a, there are four snapshots: the two blue snapshots are used for constructing a feature vector. In the training time line, the red snapshot provides labels for model induction. In the testing time line, the red snapshot is the newly observed data on which we test our model. The red testing snapshot must always have the same boundaries for fair model comparison regardless of changes to any other snapshots. We also choose to maintain the boundaries on the red snapshot in training, since varying the snapshot may cause changes in the observed prior and conditional probabilities of the link formation labels. We likewise make an effort to reflect the duration of the red snapshot in testing with the duration of the red snapshot in training to give the model the best chance at facing the same distribution on which it was trained.

Figures 7b and c demonstrate supervised learning with two temporal relations, and this requires six distinct snapshots total for training and testing. Figures 7d and e demonstrate supervised learning with three temporal relations, and this requires eight distinct snapshots. In our temporal models, we handle data granularity in two different ways. In the first, snapshot granularity remains consistent throughout the data to model. In the second, we decrease snapshot granularity exponentially with each snapshot and emphasize the most recent events by making the most recent snapshot relatively short. The depictions in Figures 7b and d are of evenly divided snapshots. Those in Figures 7c and e are of exponentially divided snapshots. In Figure 7d, we suppose that *G*_*t*−6_ is derived from the oldest available data in the stream, so we cannot extend the oldest snapshot to its full length. In such cases, it may also be wise to limit the length of the oldest testing snapshot to present the closest possible testing data to the model.

Smaller snapshots effectively place greater emphasis on individual events in the multirelational VCP method. Links from short periods and links from long periods both receive their own relations, so short periods with few events afford the contained events greater discriminative potential. Links occurring within the same snapshot are also guaranteed to occur closer together in time, which may decrease noise and increase the clarity of temporally proximate evolutionary trends. In Figures 7b and d, events that recently occurred are given the same weight as those that occurred in the more distant past. In Figures 7c and e, recent events are effectively given more weight by placing fewer, more proximate events in the same relation. In all of the depictions in Figure 7, the number of snapshots excluding the red testing snapshot is equal to the number of relations that will appear in multirelational isomorphism classes of three or four vertices like those in Figure 7.

### Longitudinal data

We draw our conclusions from a selection of four of the network data sets above. Table 7 presents some basic information about the data in a temporal context. The table also indicates the chunks into which we divided the events. For instance, we divide the longitudinal condmat data into 6 chunks, and each chunk spans 12 months. The division of data into chunks is arbitrary but represents our attempt to balance data sufficiency in each chunk with the production of sufficient chunks to allow for experiments involving variation in the number and granularity of snapshots.

**Table 7:** **Defined characteristics of selected longitudinal networks**

Network	Nodes	Edges	Chunks × Duration
condmat	17,000	110,538	6 × 12 months
dblp-collab	367,725	2,088,710	13 × 24 months
hepth-collab	8,381	40,736	6 × 24 months
huddle	4,243	993,288	11 × 3 months

**Figure 8:**
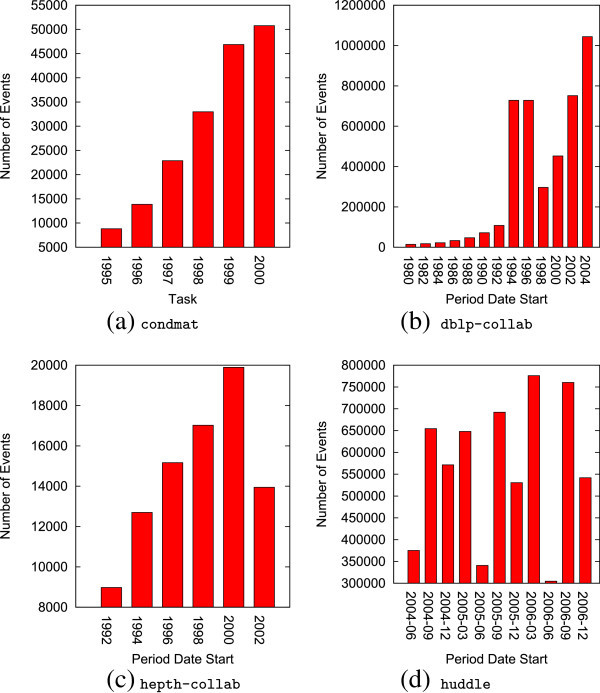
**Distribution of events by chunk.** **(a)** condmat, **(b)** dblp-collab, **(c)** hepth-collab, **(d)** huddle.

Figure 8 provides the domain and distribution of longitudinal events for each of the networks. The collaboration networks exhibit a general increase in the rate of growth. The growth in the dblp-collab network is consistent with (Ley 2002), but we note that the anomaly in 1994-1996 is inconsistent, and upon further examination of the data, we can report no explanation. The hepth-collab network data reduces in volume for 2002 due to incomplete information for that year. The huddle product copurchasing network shows a cyclical reduction during summer months, and we attribute this to the presence of fewer students on campus. There is a lesser cyclical reduction during the winter break.

### Experiments

To explore the many possibilities of encoding temporal information into multirelational representations, we perform several experiments designed to confirm or deny certain hypotheses. Areas we explore include how to divide data, whether event recency is significant, whether our method appropriately captures temporal trends or whether it is simply a surrogate for weight, and to what extent our approach scales. Performance is again determined based on predictions within *ℓ*=2 of all training data.

#### Precluding surrogacy for weight

Defining relations in terms of successive exclusive network snapshots allows the relations to offer a rudimentary encoding of weight. An alternate explanation for any performance disparities between temporally aware and unaware predictors is that the temporally aware predictors merely offer an indication of edge weight. To preclude this alternate explanation, we compute performance before and after randomly reordering events. If dividing data into snapshots and specifying multiple relations is only serving as a surrogate for weight, we expect to see approximately the same performance. If the order of the events is significant, we expect to see higher performance from temporally ordered data.

The results in Table 8 demonstrate the benefits of the temporal ordering. There is no case in which the model performs as well with randomly reordered data, and in some cases multiple relations using randomly reordered data performs worse than the unirelational baseline. This strongly refutes the hypothesis that encoding temporal information in VCPs is boosting performance merely through edge weight surrogacy.

**Table 8:** **Contrasting performance for randomly reordered data**

(a) AUROC						
**Network**	**VCP** ^**3,1,0**^	**VCP** ^**3,2,0**^	**VCP** ^**3,2,0**^ **Reordered**	**VCP** ^**4,1,0**^	**VCP** ^**4,2,0**^	**VCP** ^**4,2,0**^ **Reordered**
condmat	0.637	**0.670**	0.632	0.582	0.646	0.589
dblp-collab	0.640	**0.749**	0.682	0.695	**0.789**	0.707
hepth-collab	0.598	**0.738**	0.599	0.622	**0.732**	0.596
huddle	0.881	**0.929**	0.886	0.888	**0.926**	0.888
**(b) AUPR**						
**Network**	**VCP** ^**3,1,0**^	**VCP** ^**3,2,0**^	**VCP** ^**3,2,0**^ **Reordered**	**VCP** ^**4,1,0**^	**VCP** ^**4,2,0**^	**VCP** ^**4,2,0**^ **Reordered**
condmat	0.011917	0.010643	0.011001	0.008588	0.010103	0.009829
dblp-collab	0.007152	**0.009161**	0.008131	0.009410	**0.012389**	0.008881
hepth-collab	0.007197	**0.010280**	0.006494	0.007157	**0.009375**	0.006622
huddle	0.039394	**0.065830**	0.044412	0.046803	**0.071811**	0.046162

Columns showing performance with reordered data represent the mean performance over 10 random orderings of training data. Bold values indicate temporally ordered data outperforming randomly ordered data with statistical significance at 99.9% confidence.

#### Analyzing split points

Separating fine-grained longitudinal data into discrete, bounded units for constructing network snapshots elicits questions such as how many snapshots to use, at what temporal locations data should be divided, and whether to maintain a consistent snapshot size. We conduct a simple experiment to determine an appropriate temporal location at which to divide these data sets in the case of two snapshots. This experiment also tests the hypothesis that greater focus on recent events is beneficial, and the results lead to cogent theories about how to parameterize more snapshots.

**Figure 9:**
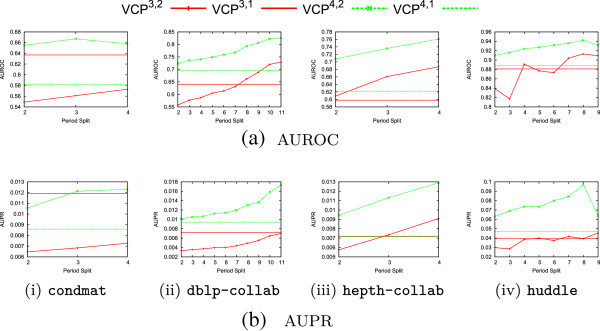
**Performance over varying temporal split locations.** **(a)** AUROC, **(b)** AUPR: (i) condmat, (ii)dblp-collab, (iii) hepth-collab, (iv) huddle.

Figure 9 shows the performance achievable at all possible splits for two snapshots given the predetermined chunk boundaries. Take *s*_*i*_ as a value in the domains of the Figure 9 sub-figures and *s*_*t*+1_ as the final chunk in the data stream as indicated in Table 7. Then in training the first snapshot includes chunks [*s*_0_,*s*_*i*_), and the second snapshot includes [*s*_*i*_,*s*_*t*−1_]. The penultimate chunk, *s*_*t*_, is reserved for training labels. For this experiment, to ensure that training and testing feature data volumes are constant, the first testing snapshot entirely subsumes the first training snapshot. In testing, the first snapshot includes chunks [*s*_1_,*s*_*i*+1_), and the second snapshot includes [*s*_*i*+1_,*s*_*t*_]. The final chunk, *s*_*t*+1_, is reserved for testing labels. The horizontal lines correspond to baselines in which supervised models are built using VCP ^*n*,1,0^, which represents no temporal discrimination.

The time-resolved multirelational VCPs almost always outperform their non-longitudinal unirelational counterparts for some split value. The only counterexample is condmat for which VCP ^3,1,0^ outperforms VCP ^3,2,0^. We additionally observe that while VCP ^4,*r*,0^ outperforms VCP ^3,*r*,0^ in all cases except condmat and nearly ties for hepth-collab AUPR and huddle AUROC, the addition of temporal discrimination into the models appears to allow VCP ^4,*r*,0^ significantly greater explanatory power across all networks. Consistent with our hypothesis regarding the significance of recency, performance increases as the second period encompasses fewer, more temporally proximate events. This confirmation is particularly stark, because excepting condmat the highest-performing split places only *s*_*t*−1_ in the second snapshot, and yet this outperforms models constructed without splitting the data.

#### Effectiveness of temporal data

Having demonstrated the importance of splitting periods and recency, we provide the comparative performance of the temporally non-resolved baseline with that of evenly and exponentially spaced snapshots in Table 9. We are interested in how much the addition of temporal information can improve the results. Gray cells indicate data sets for which there are insufficient chunks to meaningfully differentiate equal and exponential snapshot sizes with three snapshots. We consider it critical to compare the different variations using the same volume and quality of data, so we deviate slightly from the method displayed in Figure 7 in two minor ways. First, to prevent explanations of differences in performance due to variations in data, all methods use the entire quantity of historically available data for training regardless of how that data is divided into periods. In both equal and exponentially growing snapshot sizes, when there are too few or too many chunks for assignment, we select the oldest snapshot to deviate from its ideal size. This decision is based on the premise substantiated by Figure 9 that this is the snapshot of least significance to model performance. Second, to help account for potential periodic biases, especially in the huddle data set, the most recent snapshot in the exponential results starts with two rather than one underlying chunk.

**Table 9:** **Performance for longitudinal VCP predictions**

(a) AUROC - VCP ^3, ***r***^				
Network	1	2	3	3 (Exp.)
condmat	0.637	0.670	0.671	-
dblp-collab	0.640	0.749	0.770	0.779
hepth-collab	0.598	0.738	0.729	-
huddle	0.881	0.933	0.929	0.936
**(b) AUROC - VCP** ^**4,*r***^				
Network	1	2	3	3 (Exp.)
condmat	0.582	0.646	0.653	-
dblp-collab	0.695	0.789	0.807	0.817
hepth-collab	0.622	0.732	0.720	-
huddle	0.888	0.926	0.929	0.941
**(c) AUPR - VCP** ^**3,*r***^				
Network	1	2	3	3exp
condmat	0.011917	0.010643	0.010352	-
dblp-collab	0.007152	0.009161	0.009931	0.009973
hepth-collab	0.007197	0.010280	0.011131	-
huddle	0.039394	0.065830	0.067993	0.080036
**(d) AUPR - VCP** ^**4,*r***^				
Network	1	2	3	3exp
condmat	0.008588	0.010103	0.009433	-
dblp-collab	0.009410	0.012389	0.014008	0.015229
hepth-collab	0.007157	0.009375	0.009024	-
huddle	0.046803	0.071811	0.073406	0.092726

These results show that temporally aware predictors universally outperform the most directly comparable non-temporal baselines. Gains are consistently large when moving from no temporal awareness to division of time into two snapshots with VCP ^*n*,2,0^. They are less consistent moving from VCP ^*n*,2,0^ to three snapshots with VCP ^*n*,3,0^. Particularly, we note that condmat and hepth-collab do not offer significant gains or even suffer losses when moving to three snapshots. We point out the small relative volume of data in these cases and cite the curse of dimensionality. As the number of distinct temporal-structural elements increases, the amount of information contained within any one element is reduced as the data falls into increasingly many fine-grained buckets. With the voluminous dblp-collab and huddle data sets, there is still sufficient data to lend statistical credence to the greater number of features, and this is reflected by large performance gains when moving from VCP ^4,2,0^ to VCP ^4,3,0^.

### Computational challenges

The first computational challenge in highly multirelational environments relates to the time and space required to compute and store the translational subgraph-to-element vector. In our coverage of VCP theory, we described static and dynamic subgraph-to-element isomorphism class computations. Several considerations motivate the use of one or the other. Static computation gives contiguous, invertible indices for consecutive VCP elements and offers the fastest possible subgraph-to-element translations. Memoized dynamic computation, though potentially slower, also potentially decreases the amount of memory necessary to create non-contiguous but unique indices for VCP elements by only identifying and storing elements that are observed. For static computation, the space required to represent the translation from subgraphs in *G*^*n*,*r*,*d*^ to elements in VCP ^*n*,*r*,*d*^ increases in direct proportion to |*G*^*n*,*r*,*d*^|. If the VCP vector is large, then constructing the full translational vector has high cost in time and space. Dynamic computation using arbitrary scale integer indices overcomes this challenge and makes it possible to handle VCP vectors with extremely large cardinalities. For instance, we have effectively applied VCP analysis to semantic graphs in which the vector contained 7.66×10^53^ elements. The ontological consistency in the semantic graphs placed tight constraints on VCP population, and fewer than 1000 elements were actually present. Dynamic translation incurs only the cost of these elements.

The second challenge is related to allocation costs, output volume, and limitations of disk storage. Output volume grows with the size of the VCP vector. Since large networks may contain billions of edges and link prediction target pair sets may be even larger, the space necessary to represent the output of highly multirelational VCP analysis is problematic. As we showed with Figure 4, even in worst-case networks, the number of non-zero elements in the vector may be small even when its cardinality is large. Even before the value of *r* grows so that subgraph computation and space for translation vectors is constrained, the cost of repeatedly allocating and deallocating the space for large VCP vectors becomes troublesome. The solution is to maintain an associative data structure of vector elements that exist for a given vertex pair, which will often be a small fraction even of the subset of elements existing in the graph. Rather than outputting the full vector, we output a list of (index,count) pairs, precluding the necessity of prohibitively allocating space for every element and substantially reducing the cost of printing and storing the output. From a classification perspective, this offers zero-cost filtering of VCP elements for which there is no representative membership in the training set, sacrificing no information.

These solutions make VCP analysis feasible for nearly all combinations of *n*, *r*, and *d* likely to appear in real-world data. We next turn to computational challenges involved in performing classification with VCP output. The dblp-collab network has 1.07×10^7^ potential *ℓ*=2 links that populate 12,436 VCP ^4,2,0^ elements with non-zero values. The huddle network has 7.29×10^6^ potential *ℓ*=2 links that populate 16,635 VCP ^4,2,0^ elements. Assuming 8-byte feature representations for 10 million training instances, typical of *ℓ*=2 link prediction in moderately sized social networks, the in-memory data set footprint for fully populated VCP ^4,3,0^ is 5.2 TB.

This is strictly an engineering problem, and many solutions are readily available. Information density in VCP elements varies widely, so dimensionality reduction and feature selection are well-suited to reducing data volume. These methods must be exercised strategically, however. We cannot load 5.2 TB of data into memory to run such procedures. Processing the training data file one feature at a time requires reading more than 100 PB of I/O. Reading the file once and using process substitution requires thousands of simultaneous active processes and completely overwhelms the scheduler. We can first reduce data volumes to arbitrary levels by undersampling, which also reduces the number of pairs for which VCP vectors must be computed. In dblp-collab, when the negative class is reduced to a 3:1 ratio, only 88,744 instances remain. This ratio, which was practicable enough to obtain our results, makes many dimensionality reduction and feature selection schemes feasible, and even inefficient implementations of classification algorithms such as naïve Bayesian classifiers and decision forests can process such volumes of data in tractable time. Compression methods that are lossless with respect to class entropy, such as binary quantization of VCP elements for which all non-zero values correspond to only a single class, can also make data set storage and processing more efficient.

**Figure 10:**
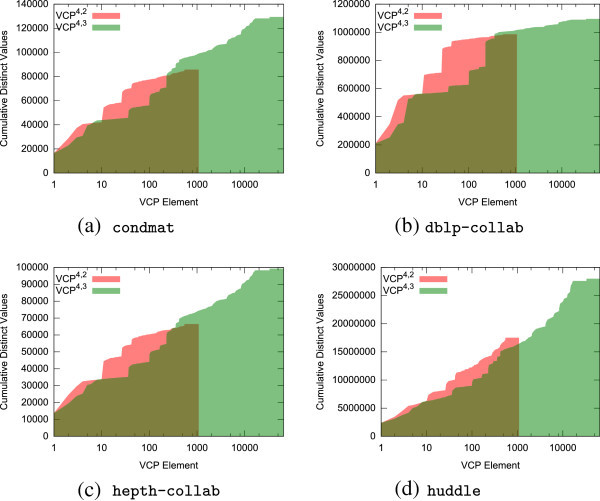
**Scaling trends in distinct values of VCP elements.** **(a)** condmat, **(b)** dblp-collab, **(c)** hepth-collab, **(d)** huddle.

An even more scalable solution results from a consequence of the curse of dimensionality. We observe that as the number of VCP elements increases for a given graph, the number of distinct non-zero values decreases within each element due to increasing information sparsity. Figure 10 shows the cumulative distribution of distinct values across VCP ^4,2,0^ and VCP ^4,3,0^. In all cases, though there are more than 60 times as many features with four relations, the sum of the cardinalities of the sets of distinct values across the features is less than doubled. We use this fact to implement a single-pass ordered map representation of each feature in the data set that associates values with their frequency of occurrence. The representation is highly compact: the huddle data set, which requires more than 80 GB of memory in matrix representation, requires only 1.4 GB in the map. This method effectively implements a fast, non-contiguous counting sort that allows for nearly instantaneous computation of feature selection methods such as information gain. Information gain is fast, but the well-known limitation of all filtering methods of feature selection is that they fail to consider feature subsets and thus cannot consider feature orthogonality, correlation, and dependencies. Nonetheless, when we constructed models using only the 1000 strongest VCP elements, we were able to reduce classifier training times by a factor of 100, reduce memory requirements by a factor of 50, and achieve AUROC and AUPR performance within 2% of that achieved by models bestowed with all VCP elements.

## Conclusions

VCP is a new method for link analysis with solid theoretical roots. We presented evidence of its utility in some applications here, but there are many possible applications. It is useful for post hoc analysis of classification output and comparative analysis of network link structure, and it competes effectively with existing link prediction methods, often outperforming them by wide margins. In well-established networks with past observational data, VCP can serve as a sensitive change detection mechanism for tracking the evolving link formation process. In addition to link prediction and link analysis for the purpose of network growth modeling, VCP can be used for link or vertex pair clustering. Its ability to handle multiple relations naturally extends its utility into many domains and offers an alternative to the practice of combining or discarding edge types or edge directionality. We showed how VCP analysis can be useful in incorporating temporality into link prediction with a multirelational encoding, and this offered improvements another factor greater than what we already obtained with unirelational VCP predictions.

Optimized implementations of VCP ^3,*r*,*d*^ and VCP ^4,*r*,*d*^ algorithms are available at https://github.com/rlichtenwalter/vcp. The repository includes subgraph-to-element mapping software, which generates static subgraph-to-element mappings for 3≤*n*≤8,1≤*r*≤8,*d*∈{0,1} and dynamic subgraph-to-element mappings for any values of *n*, *r*, and *d*. Algorithms for VCP ^3,1,*d*^ and VCP ^4,1,*d*^ are also integrated into the LPmade link prediction framework and are available at http://mloss.org/software/view/307/. Most of the data sets are publicly available elsewhere, but we have also published all public data sets at http://nd.edu/~dial/vcp/.

## Appendix A

In the original exposition of VCP analysis in (Lichtenwalter and Chawla 2012), we provided the equation for the number of subgraphs in universes *G*^*n*,*r*,0^ given by the tuples (*n*,*r*) and deliberately excluded a target prediction relation between *v*_*s*_ and *v*_*t*_, from consideration. The equation appeared thus:
16

The edge *e*_*s*,*t*_ received special treatment for two reasons. First, particular analytical domains offer additional knowledge about this edge. For instance, in link prediction we will usually be interested in analyzing only vertex pairs (*v*_*s*_,*v*_*t*_) for which *e*_*s*,*t*_∉*E*. In link clustering, we are likely interested in *e*_*s*,*t*_∈*E*. Second, for a given (*v*_*s*_,*v*_*t*_), only 2^−*r*^ of all elements will be non-zero if the edge does not receive special treatment, because *e*_*s*,*t*_ takes a constant form in all embeddings. The information about the nature of *e*_*s*,*t*_ is thus more succinctly described, when it is desired at all, as a single integer taking values [ 0,2^(*d*+1)*r*^−1], which is sufficient to fully define the edge.

The treatment in Section “Vertex collocation profiles” gains greatly in theoretical consistency and mathematical coherence by not considering *e*_*s*,*t*_ in a special way. Additionally, goals such as tensor analysis of VCP vectors may gain from the verbose representation. The provided VCP code implementations are fully aligned with the presentation in Section “Vertex collocation profiles”. When one is using VCP for link prediction specifically, it may be convenient to alter this scheme by effectively shifting the lexicographical ordering of the edges so that *e*_*s*,*t*_ is the edge of highest value, as presented in (Lichtenwalter and Chawla 2012). By placing that edge in the *most* significant position, in the common link prediction case where no edge exists between *v*_*s*_ and *v*_*t*_, unpopulated elements are grouped in the second half of the vector. It is trivial to modify our code to achieve this by adjusting the value of enum constants and using a modified subgraph-to-element mapping. Then one may truncate the vector either prior to outputting it in code or afterward with, for instance, the Unix cut command.

## Appendix B

An anonymous reviewer has suggested presenting a more intuitive explanation of subgraph addressing. We maintain our primary explanation in the main body for its rigor, its ties to spectral graph theory, and its mathematical clarity in the permutation approach to identifying isomorphisms. We recognize, however, that the following explanation is more intuitive for many. We note that this only covers subgraph addressing and not VCP addressing. We thank the anonymous reviewer for providing the following explanation, which we have adjusted somewhat.

Our goal is to map a subgraph to an index in the subgraph universe *G*^*n*,*r*,*d*^, which has cardinality . Let the vertices of the subgraph be labeled *v*_1_=*v*_*s*_,*v*_2_=*v*_*t*_,*v*_3_,…,*v*_*n*_. Assume the graph is undirected, so *d*=0. Then there are  possible edges. Without loss of generality, we assume that these edges are lexicographically ordered and that the lower index is always first so that edge 1 is (*v*_*s*_,*v*_*t*_), edge 2 is (*v*_*s*_,*v*_3_), and edge *p* is (*v*_*n*−1_,*v*_*n*_). The index into the VCP vector is represented in binary with most significant bits first as:


Each edge is an *r*-bit value representing which relationships are present. For example, if there are *r*=3 possible edge types, then the presence of the first and second is represented as 011, and the presence of none is represented as 000.

If the network is directed, so *d*=1, then the number of possible edges doubles since we separately consider (*v*_*i*_,*v*_*j*_) and (*v*_*j*_,*v*_*i*_). We maintain the same lexicographical ordering on edges, but we admit both directions. So, the representation is:


Clearly the number of bits is . Since 2^*d*^=*d*+1 for *d*∈{0,1}, we can see that this is the correct number of indices.
